# Association between personality traits and symptoms of depression and anxiety via emotional regulation and distress tolerance

**DOI:** 10.1371/journal.pone.0306146

**Published:** 2024-07-18

**Authors:** Paula Aguirre, Yanina Michelini, Adrian J. Bravo, Ricardo Marcos Pautassi, Angelina Pilatti

**Affiliations:** 1 Facultad de Psicología, Universidad Nacional de Córdoba, Córdoba, Córdoba, Argentina; 2 Instituto de Investigaciones Psicológicas, IIPsi-CONICET-UNC, Córdoba, Córdoba, Argentina; 3 Department of Psychological Sciences, William & Mary, Williamsburg, Virginia, United States of America; 4 Instituto de Investigación Médica M. y M. Ferreyra, INIMEC-CONICET-UNC, Córdoba, Córdoba, Argentina; Beijing University of Technology, CHINA

## Abstract

The Big Five personality traits have shown associations with symptoms of depression and anxiety among college students, but it is unclear which factors mediate these relationships. Past research suggests that psychological distress is closely related to difficulties in affect regulation (e.g., low distress tolerance). Therefore, the present study examined the associations between personality traits and depression and anxiety via emotion regulation and distress tolerance. Participants were 694 (81.4% females; Mean age = 23.12 [SD 2.75]) Argentinian college students who completed an online survey examining mental health and personality variables. A sizeable percentage of students endorsed moderate to severe symptoms of depression (45.1%) or anxiety (25.9%). Utilizing path analyses, we found that appraisal, a dimension of distress tolerance, atemporally mediated the association between emotional stability and symptoms of depression/anxiety (i.e., higher levels of emotional stability → higher appraisal distress tolerance → fewer symptoms of depression/anxiety). Further, expressive suppression (a dimension of emotion regulation) significantly mediated the associations between personality traits (i.e., agreeableness and extraversion) and symptoms of depression (higher levels of agreeableness/extraversion → lower use of expressive suppression → fewer symptoms of depression). Taken together, the results suggest that higher levels of emotional stability, extraversion and agreeableness could protect students from the development of symptoms of depression/anxiety via lower maladaptive emotion regulation strategies and higher distress tolerance (particularly appraisal). These findings highlight the relevance of intervention strategies specifically tailored to improve distress tolerance and emotion regulation for those students undergoing mental health problems.

## Introduction

Emerging adulthood, the transitional period from adolescence to young adulthood, involves social and economic instability [1] and is associated with increased vulnerability for the development of mental health problems [2]. During these years, many individuals also transition from high school to college, which is often associated with a decrease in psychosocial adjustment and wellbeing [3, 4]. A study with college students from eight countries found that almost 40% had suffered from mental health problems in the previous year [5]. In Argentina, the country where the present study was conducted, research focused on mental health problems in college students is limited. However, findings from a recent study indicated that 72% of the Argentinian students sampled reported psychological distress [6]. In another study, researchers found that 44.7% of Argentinian college students met a threshold for depression symptoms criteria and may warrant further investigation regarding depression symptom presentation [7]. Moreover, Mezquita et al. [8] found that 25% and 33% of the Argentinian students included in their sample reported symptoms of anxiety or depression, respectively.

The factors that determine the development of mental health problems are still under investigation, yet personality traits provide different levels of risk [9]. One of the most widely accepted models for the study of personality is the *Big Five Model*, which proposes five personality traits [10]: openness to experience (i.e., intellectual curiosity, imagination), extraversion (i.e., sociability, activity levels, assertiveness), agreeableness (i.e., propensity to empathy, collaboration, cooperation), conscientiousness (i.e., propensity to planning, impulse control, organization) and neuroticism or, inversely, emotional stability [8, 11]. High levels of neuroticism have been associated with greater symptoms of depression or anxiety [8, 9, 11–14]. In line with this, a study with college students from 3 countries found that symptoms of internalizing mental health problems (i.e., depression, anxiety and somatic distress) were associated with high levels of neuroticism and, although smaller in strength, significant negative correlations were also found between symptoms of depression and anxiety and extraversion, agreeableness, and conscientiousness [8]. Moreover, a meta-analysis by Kotov et al. [9] examined correlational studies between personality traits and various mental health problems and found that a combination of high neuroticism, low conscientiousness, and low extraversion were associated with internalizing mental health problems. These results suggest that the traits most associated with internalizing mental health problems are neuroticism, extraversion, and conscientiousness.

It has been postulated that depressive and anxiety disorders arise from difficulties in emotion regulation (ER) [15]. Gross and Thompson [16] defined ER as the set of automatic or controlled cognitive processes through which people attempt to modify the occurrence, magnitude, duration, or expression of their emotional responses. Gross and John [17] proposed two ER strategies: cognitive appraisal (i.e., cognitively reconstructing a situation that may trigger an emotional response in a way that reduces its emotional impact) and expressive suppression (i.e., attempts to inhibit the behavioral expression of an emotion). A more frequent use of cognitive appraisal is associated with the experience of more positive and less negative emotions [17] as well as less symptoms of depression [18, 19]. Two meta-analyses found that expressive suppression is associated with reduced positive affect [20] and with greater symptoms of depression and anxiety [18].

Personality traits are also associated with ER strategies. Specifically, a meta-analysis found that greater levels of neuroticism and lower levels of extraversion, openness to experience, agreeableness, and conscientiousness were associated with greater use of maladaptive strategies and lower use of adaptive strategies [21]. Consistent with these results, it has been reported that ER strategies have a mediating role in the relationship between neuroticism and depression [22, 23], anxiety [22], psychotic symptoms [24], and life satisfaction [25]. ER strategies may also mediate the association between other personality traits and mental health problems. High extraversion is associated with less anxiety via a greater use of cognitive reappraisal [22], and with greater levels of life satisfaction via reduced use of expressive suppression [25].

Another key component of affect regulation is distress tolerance (DT), the real or perceived ability to withstand negative and uncomfortable psychological states [26]. This construct includes four components: tolerance (i.e., the perceived tolerability and aversiveness of distress); appraisal (i.e., the subjective evaluation of distress as unacceptable or shameful); absorption (i.e., the tendency to have one’s attention absorbed by the negative event that’s causing distress); and regulation (i.e., the magnitude of the efforts made to avoid or attenuate the experience of distress). Low levels of DT are associated with greater depression [27–30] and anxiety symptoms [27, 30, 31]. DT is also negatively associated with neuroticism [32–34] and positively associated with extraversion and conscientiousness [32]. Moreover, DT seems to mediate the association between personality traits like neuroticism or extraversion and mental health problems [30].

### The present study

Some interventions targeting DT [35–38] have shown promising results, which had led to a growing interest in understanding the role of DT in the association between distal variables (such as personality traits) and psychopathology [39]. Therefore, the study of these associations will help identify the mechanisms involved in the development and maintenance of internalizing mental health problems in college students. To our knowledge, research examining the indirect association between personality traits and symptoms of anxiety and depression via ER and DT in college students is limited [25, 30], particularly in South-America.

**Fig 1:**
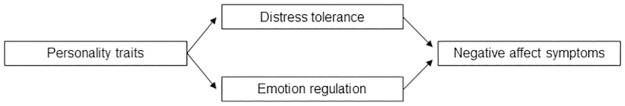
Analyzed conceptual model.

Noteworthy, culture plays a crucial role in the experience and evaluation of distress and in the regulation of emotions [40, 41]. Culture signals which behaviors align, or not, with culturally supported values and, therefore, affects the probability of their occurrence. Illustrating this, certain cultural values (e.g., independence vs. interdependence) affect which emotion regulation strategies are used more frequently; and also modulate the association between these strategies and psychological wellbeing [42]. There is a need to investigate the association between personality traits, emotion regulation, distress tolerance and psychological distress in different cultural scenarios. It is possible that these associations could be affected by cultural patterns idiosyncratic of the Argentinean context, the South American country where the present study was conducted. Further, data collection for the present study took place during the COVID-19 pandemic period. Other sociodemographic factors, like socioeconomic status (SES), may influence these associations [43, 44]. Thus, the present study examined, in a sample of Argentinian college students: a) the occurrence of symptoms of depression and anxiety among university students, and b) the indirect associations of the personality traits proposed by the Big Five model and SES with symptoms of depression and anxiety, via ER and DT (Fig 1).

We hypothesized that: 1) lower emotional stability (i.e., higher neuroticism) would be indirectly associated with more symptoms of depression and anxiety via ER (i.e., higher use of expressive suppression and lower use of cognitive reappraisal) and lower DT, 2) lower extraversion would be indirectly associated with more symptoms of depression and anxiety via ER (i.e., higher use of expressive suppression and lower use of cognitive reappraisal) and lower DT, and 3) lower conscientiousness would be indirectly associated with more symptoms of depression and anxiety via ER (i.e., higher use of expressive suppression and lower use of cognitive reappraisal).

## Materials and methods

### Sample

Participants were recruited using a convenience sampling procedure. Specifically, we disseminated a flyer through online social networks (i.e., Instagram, Facebook, and Twitter) and e-mail listings. The flyer asked for college students, aged 17–30 years old, enrolled in three public universities (i.e., two located in Cordoba and one in Ciudad Autónoma de Buenos Aires [*Autonomous City of Buenos Aires*], the two most populated cities of Argentina). Approximately 80% of the students at these universities belong to families of medium or high SES [45].

Although 1,339 individuals fully or partially completed the survey, we excluded 645 cases for one of the following reasons: 1- only provided socio-demographic information (n = 67), 2- were enrolled in non-targeted universities (n = 42), 3- were duplicated responses (n = 64), 4- failed in at least three of five attention check questions (n = 48 [see the procedures section]), 5- were not college students (dropped out of college or already graduated; n = 29), 6- were 31 or older (n = 135), or 7- did not complete measures assessing the dependent variables (n = 260). The final analytic sample was comprised of 694 participants (81.4% females, 18.6% males [based on participants’ responses to sex assigned at birth]; Mean age = 23.12 [SD 2.75]). Mean perceived SES (from 1 = *very poor* to 10 = *wealthy*) was 5.86 (SD 1.75). While the majority of the participants reported being unemployed (62.8%), 17.6% reported working up to 20 hours/week, 15.9% up to 40 hours/week, and 3.7% reported working more than 41 hours/week. The majority (97.6%) did not have kids. Regarding course year, 9.2% were freshman, 13.4% were sophomore, 25.2% junior, 20% senior, 13% and 19% were in their fifth or sixth/seventh year of college, respectively. More than half of the participants (60.1%) were enrolled in one of the two universities of Cordoba and the remaining 39% was enrolled in the university from the Autonomous City of Buenos Aires.

### Procedure

Participants completed an online survey (*LimeSurvey*) that took ~50 minutes to complete. The survey had several attention checks that requested participants to select a particular response option (e.g., ‘for this statement, please select the option *disagree’*). To avoid missing responses, participants received electronic prompts for each missing response. Those who completed the survey were eligible to participate in a raffle (a cash prize equivalent to ≈30 US Dollars at the moment of data collection and 20 gift cards [each equivalent to ≈10 US Dollars at the moment of data collection] to use in a bookstore). Although we did not ask for identifiable information, participants were invited to provide their emails to identify duplicates and to inform participants if they won one of the prizes. Data was collected between March and April 2021.

### Measures

For all psychometric measures, composite scores were created by summing items (reverse-coding of items were conducted when appropriate) such that higher scores indicate higher levels of the construct.

#### Personality

We used the Spanish version [46] of the Big Five Personality Trait Short Questionnaire (BFPTSQ) [11]. This is a 50-item self-report measure that assesses five personality domains (10 items per domain): openness (e.g., *I see myself as someone who is curious about many different things*), extraversion (e.g., *I see myself as someone who likes to talk*, *expresses his/her opinion*), emotional stability (or low neuroticism; e.g., *I see myself as someone who is emotionally stable*, *not easily upset*), agreeableness (e.g., *I see myself as someone who is considerate and kind to almost everyone*), and conscientiousness (e.g., *I see myself as someone who plans things that need to be done and follows through the plans*). Participants rate, on a 5-point response scale (from 0 = *totally disagree* to 4 = *totally agree*), their level of agreement with each statement. This measure was previously used with college students from Argentina [47] and past research supported the BFPTSQ as an adequate measure to assess these personality domains in Spanish- and English-speaking college students [8]. Cronbach’s alphas were, in the study that validated the Spanish version [46], between .75 and .87. In the present study, Cronbach’s alphas were as follows: .83 (Openness), .87 (Extraversion), .87 (Emotional stability), .73 (Agreeableness) and .84 (Conscientiousness).

#### Emotion regulation strategies

We used the Spanish version [48] of the Emotional Regulation Questionnaire (ERQ) [17] to assess cognitive reappraisal (6 items; e.g., *I control my emotions by changing the way I think about the situation I’m in*) and expressive suppression (4 items; e.g., *I control my emotions by not expressing them*), the two dimensions of emotion regulation strategies assessed by the ERQ. Participants rated their level of agreement with each statement (from 1 = *strongly disagree* to 7 = *strongly agree*). This version was used with college students from Argentina [49]. In the validation study for the Spanish version [48], Cronbach’s alphas were .79 (Cognitive reappraisal) and .75 (Expressive suppression). In the present study, Cronbach’s alphas were .81 (Cognitive reappraisal) and .80 (Expressive suppression).

#### Depression symptoms

We used the Spanish version of the Patient Health Questionnaire-9 (PHQ-9), a self-report measure that assesses severity of depression. The nine items are based on the DSM-V [50] criteria for the diagnosis of major depressive disorder. Participants reported how often (from 0 = *not at all* to 3 = *nearly every day*) they experienced the problems described by each statement, in the past month (e.g., *Feeling down*, *depressed*, *or hopeless*). The “Spanish for Spain” version was used in this study, which is a public domain measure [50]. For descriptive purposes, participants were classified according to the degree of severity of depression: 0–4 [no depression], 5–9 [mild depression], 10–14 [moderate depression], 15–19 [moderately severe depression], 20–27 [severe depression]. Psychometric analyses conducted with this same sample of participants supported the adequate reliability of the scores and provided evidence of internal and criterion-related validity [51]. The scale featured adequate reliability in the present study (α = .87).

#### Generalized anxiety symptoms

We used the Spanish version [52] of a public domain measure of the Severity Measure for Generalized Anxiety Disorder-Adult [50]. This is a 10-item self-report measure in which participants report how often (from 0 = *never* to 4 = *always;* scores can range between 0 and 40) they have experienced the problems described by each statement, in the past month (e.g., *Avoided*, *or did not approach or enter*, *situations about which I worry*). Vidal-Arenas et al. [52] found adequate reliability and evidence of internal, convergent, and criterion-related validity. For descriptive purposes, participants were classified according to the degree of severity of GAD: 0–4 [no GAD], 5–14 [mild GAD], 15–24 [moderate GAD], 25–34 [severe GAD], 35–40 [extreme GAD]. Psychometric analyses conducted with this same sample of participants supported the adequate reliability of the scores and provided evidence of internal and criterion-related validity. The scale featured adequate reliability in the present study (α = .86).

#### Distress tolerance

We used the Spanish version [30] of the Distress Tolerance Scale (DTS) [26]. The DTS is a 15-item self-report measure developed to assess the perceived ability to tolerate psychological distress within four domains: tolerance (3 items; e.g., *Feeling distressed or upset is unbearable to me*), appraisal (6 items; e.g., *My feeling of distress or being upset are not acceptable*), absorption (3 items; e.g., *When I feel distressed or upset*, *all I can think about is how bad I feel*), and regulation (3 items; e.g., *I’ll do anything to stop feeling distressed of upset*). Participants rated, on a 5-point scale (from 1 = *strongly agree* to 5 = *strongly disagree*), their level of agreement with each statement. This version was previously used with college students from Argentina and showed adequate reliability (between α = .73 and α = .84) for each dimension [53] and for the total score [49]. In the present study, Cronbach’s alphas were as follows: .81 (Tolerance), .82 (Appraisal), .85 (Absorption), and .74 (Regulation).

### Ethics statement

The procedures were approved by the institutional review board of the participating university and endorsed the ethical guidelines for human research of the American Psychological Association, the Declaration of Helsinki, and the National Law 25.326 for the Protection of Personal Data. Prior to participating, all individuals were provided with detailed information about the aims of the study, the procedures involved, the confidentiality for the data handling, and the voluntary nature of their participation. Participants provided informed consent by actively selecting a mandatory checkbox stating "I agree to participate in the study”. The online survey platform was designed so that participation and data submission were only possible after participants explicitly provided their informed consent by clicking on the designated button. Since all participants were aged 17 and above, informed consent was obtained directly from the participants themselves, as they were considered legally competent to provide consent for their own participation, as per Argentina’s legal framework. It is important to note that, according to this framework, consent from parents or a guardian was not required for participants in this age group.

### Data analysis

First, we summarize the percentage of students that fall into each category of severity of depression and GAD. Then, we utilized path analysis to examine the associations between personality traits and negative affect problems via DT and ER following a two-step procedure. We also included SES as a distal variable. Initially, we randomly divided the sample into two halves. With the first half (n = 347, 81.7% female), we examined the bivariate correlations between perceived SES, personality traits, components of DT, dimensions of ER, and symptoms of psychological distress (i.e., GAD and depression symptoms) Afterwards, we examined a fully saturated model where SES and all five personality traits had paths estimated on each dimension of DT, ER, and symptoms of depression/GAD. This step aimed to explore statistically significant direct and indirect associations between personality traits and depression symptoms/GAD via DT and ER. Subsequently, with the second half of the sample (n = 347, 81.3% female), we tested a model that only included the statistically significant paths identified in the first step. This approach was chosen to initially assess the number of relevant variables (first step) and subsequently reduce the number of paths estimated in the second step (reduced model). Given the cross-sectional nature of our data, our study investigated the atemporal mediation [54] of DT and ER in the links between personality traits and negative affect problems (i.e., GAD and depression symptoms). Separate models were conducted for depression symptoms and GAD. Total, direct, and indirect associations were estimated using bias-corrected bootstrapped estimates [55]. The statistical significance of the associations was determined by 95% bias-corrected bootstrapped confidence intervals (CIs) not containing zero. The descriptive and correlation analyses were conducted with SPSS 23 and the path analyses was conducted with *Mplus 8*.*4* [56].

## Results

### Descriptive results

Descriptive results regarding severity of depression and GAD symptoms for the total sample and by sex are presented in Table 1. Nearly half of the sample (45.1%) reported at least a moderate severity of depression symptoms, and this was higher in women compared to men (46.9% versus 34.4%). Around 1 in 4 students (25.9%) reported at least moderate GAD symptoms. The occurrence of more severe levels of GAD was higher in women (26%) than in men (21.3%).

**Table 1:** Severity of depression and GAD symptoms for the total sample and by sex.

	Total	Sex	
	%(n)	Female %(n)	Male %(n)
**Depression**			
**No depression**	20.7 (144)	18.7 (103)	31.1 (38)
**Mild**	34.1 (237)	34.4 (190)	34.4 (42)
**Moderate**	23.2 (161)	24.5 (135)	16.4 (20)
**Moderately Severe**	14.1 (98)	14.1 (78)	12.3 (15)
**Severe**	7.8 (54)	8.3 (46)	5.7 (7)
**GAD**			
**No GAD**	21.4 (146)	18.8 (104)	32.2 (39)
**Mild**	52.7 (360)	53.3 (294)	46.3 (56)
**Moderate**	20.9 (143)	21.2 (117)	17.2 (21)
**Severe**	4.4 (30)	4.3 (24)	3.3 (4)
**Extreme**	0.6 (4)	0.5 (3)	0.8 (1)

*Note*. GAD: Generalized Anxiety Disorder.

### Bivariate results

Bivariate correlations between the variables (SES, personality traits, DT, ER, and negative affect problems) conducted with the first half of the sample (i.e., step 1) are presented in Table 2.

**Table 2:** Bivariate correlations among study variables in the step-1 sample.

	1	2	3	4	5	6	7	8	9	10	11	12	13
1. SES													
2. Openness	.06												
3. Extraversion	.08	**.29**											
4. Agreeableness	.08	**.20**	**.18**										
5. Conscientiousness	**.17**	.07	**.19**	**.17**									
6. Emotional Stability	**.16**	**.16**	**.32**	**.21**	**.29**								
7. DT Tolerance	.04	.09	**.15**	**.11**	**.19**	**.44**							
8. DT Absorption	**.14**	.08	**.23**	**.12**	**.24**	**.59**	**.64**						
9. DT Appraisal	**.18**	**.16**	**.26**	**.17**	**.24**	**.56**	**.53**	**.61**					
10. DT Regulation	.03	.06	-.03	.03	.06	**.12**	**.35**	**.15**	**.30**				
11.Cognitive Reappraisal	.00	**.18**	**.21**	**.13**	**.17**	**.30**	.10	**.26**	**.23**	-.07			
12. Expressive Suppression	.01	**-.15**	**-.39**	**-.31**	-.06	**-.13**	-.07	**-.12**	**-.20**	-.02	.08		
13. Depression Symptoms	**-.22**	-.02	**-.27**	**-.20**	**-.33**	**-.54**	**-.37**	**-.47**	**-.48**	-.07	**-.16**	**.24**	
14. GAD Symptoms	**-.19**	.05	**-.17**	-.10	**-.24**	**-.50**	**-.37**	**-.50**	**-.52**	**-.13**	-.10	**.15**	**.72**

*Note*. Significant correlations (*p* ≤ .05) are in **bold** typeface for emphasis. SES = Socioeconomic status. DT = Distress tolerance. GAD = Generalized anxiety disorder.

### Fully saturated models

A summary of the total, indirect and direct associations of SES and personality traits on negative affect problems via DT dimensions and ER strategies for each fully saturated model is presented in Table 3.

**Table 3:** Summary of total, indirect, and direct effects of the saturated models.

Outcome variables:	*Depression symptoms*		*GAD symptoms*	
Predictor variable: *SES*	β	95% CI	β	95% CI
Total	**-.108**	**-.192, -.037**	**-.099**	**-.196, -.001**
Total indirect^a^	-.005	-.039, .029	-.030	-.069, .009
DT Tolerance	.003	-.008, .014	.000	-.008, .008
DT Absorption	-.003	-.015, .008	-.006	-.024, .011
DT Appraisal	-.012	-.029, .005	-.021	-.048, .005
DT Regulation	.001	-.008, .009	.000	-.006, .006
Cognitive Reappraisal	.000	-.008, .007	-.005	-.017, .007
Expressive Suppression	.007	-.009, .023	.003	-.006, .012
Direct	**-.103**	**-.186,—.020**	-.068	-.161, .024
Predictor variable: *Openness*	β	95% CI	β	95% CI
Total	**-.118**	**.027, .209**	**.137**	**.035, .239**
Total indirect^a^	-.002	-.036, .032	.000	-.043, .042
DT Tolerance	-.001	-.011, .009	.000	-.007, .007
DT Absorption	.002	-.010, .013	.003	-.015, .022
DT Appraisal	-.007	-.024, .010	-.013	-.040, .014
DT Regulation	.004	-.007, .015	.001	-.009, .010
Cognitive Reappraisal	.001	-.011, .012	.008	-.007, .023
Expressive Suppression	.000	-.015, .016	.000	-.007, .008
Direct	**.006**	**.033, .206**	**.138**	**.044, .231**
Predictor variable: *Extraversion*	β	95% CI	β	95% CI
Total	**-.108**	**-.199, -.017**	-.030	-.134, .074
Total indirect^a^	**-.069**	**-.119, -.018**	-.038	-.095, .019
DT Tolerance	.000	-.009, .010	.000	-.006, .006
DT Absorption	-.004	-.018, .009	-.008	-.029, .012
DT Appraisal	-.009	-.027, .009	-.016	-.046, .013
DT Regulation	-.006	-.019, .008	-.001	-.013, .011
Cognitive Reappraisal	.001	-.009, .010	.006	-.007, .019
Expressive Suppression	**-.050**	**-.088, -.012**	-.019	-.057, .020
Direct	-.039	-.133, .055	.008	-.090, .106
Predictor variable: *Agreeableness*	β	95% CI	β	95% CI
Total	-.070	-.163, .024	-.001	-.105, .104
Total indirect^a^	-.040	-.080, .000	-.016	-.064, .032
DT Tolerance	-.001	-.010, .008	.000	-.006, .007
DT Absorption	.002	-.010, .014	.003	-.016, .022
DT Appraisal	-.005	-.020, .011	-.008	-.034, .018
DT Regulation	.000	-.009, .009	.000	-.007, .007
Cognitive Reappraisal	.000	-.006, .006	.003	-.008, .013
Expressive Suppression	**-.036**	**-.066, -.007**	-.014	-.042, .015
Direct	-.030	-.123, .064	.015	-.089, .120
Predictor variable: *Conscientiousness*	β	95% CI	β	95% CI
Total	**-.164**	**-.257, -.071**	-.095	-.196, .007
Total indirect^a^	-.014	-.046, .018	-.024	-.064, .017
DT Tolerance	-.005	-.017, .007	.000	-.011, .011
DT Absorption	-.007	-.021, .006	-.014	-.033, .005
DT Appraisal	-.010	-.028, .008	-.019	-.046, .009
DT Regulation	.002	-.008, .013	.000	-.007, .008
Cognitive Reappraisal	.001	-.008, .009	.006	-.007, .019
Expressive Suppression	.006	-.010, .022	.002	-.007, .011
Direct	**-.150**	**-.241, -.058**	-.071	-.171, .029
Predictor variable: *Emotional Stability*	β	95% CI	β	95% CI
Total	**-.448**	**-.532, -.365**	**-.474**	**-.566, -.381**
Total indirect^a^	**-.150**	**-.224, -.075**	**-.218**	**-.297, -.140**
DT Tolerance	-.030	-.079, .019	.001	-.051, .053
DT Absorption	-.055	-.129, .018	**-.103**	**-.174, -.033**
DT Appraisal	**-.076**	**-.137, -.014**	**-.135**	**-.198, -.72**
DT Regulation	.007	-.008, .022	.001	-.014, .016
Cognitive Reappraisal	.001	-.020, .023	.017	-.009, .043
Expressive Suppression	.002	-.013, .018	.001	-.007, .009
Direct	**-.299**	**-.409, -.188**	**-.255**	**-.366, -.145**

*Note*. SES = socioeconomic status. DT = Distress Tolerance. GAD = Generalized Anxiety Disorder. Significant associations (*p* < .05) are in bold.

^a^ Reflects the combined indirect associations within the model

#### Depression model

SES, conscientiousness, and emotional stability had direct negative associations with severity of depression, while openness had a direct positive association. Additionally, extraversion and agreeableness had indirect negative associations with depression via expressive suppression (i.e., ER strategy), and emotional stability had an indirect negative association with depression symptoms via appraisal (i.e., component of DT). The model fit indices were: χ^2^ (63) = 994.097, *p* = .000, CFI = 1.000, TLI = 1.000, RMSEA = 0.000, SRMR = 0.000.

#### GAD model

Only two personality traits showed direct associations with severity of GAD: openness was positively associated with GAD symptoms, while emotional stability was negatively associated. Additionally, emotional stability had indirect negative associations with GAD symptoms via appraisal and absorption (i.e., components of DT). The model fit indices were: χ^2^ (63) = 975.466, *p* = .000, CFI = 1.000, TLI = 1.000, RMSEA = 0.000, SRMR = 0.000.

### Reduced models

A summary of the total, indirect and direct associations of SES and personality traits on negative affect problems via DT dimensions and ER strategies for each dependent variable is presented in Table 4.

**Table 4:** Summary of total, indirect, and direct effects of the reduced models.

**Outcome variable**:	***Depression symptoms***	
Predictor variable: *SES*	β	95% CI
Total	-.055	-.134, .024
Total indirect^a^	-	-
Direct	-.055	-.134, .024
Predictor variable: *Openness*	β	95% CI
Total	-.014	-.101, .072
Total indirect^a^	-	-
Direct	-.014	-.101, .072
Predictor variable: *Extraversion*	β	95% CI
Total	**-.060**	**-.100, -.020**
Total indirect^a^	**-.060**	**-.100, -.020**
Expressive Suppression	**-.060**	**-.100, -.020**
Predictor variable: *Agreeableness*	β	95% CI
Total	**-.025**	**-.047, -.004**
Total indirect^a^	**-.025**	**-.047, -.004**
Expressive Suppression	**-.025**	**-.047, -.004**
Predictor variable: *Conscientiousness*	β	95% CI
Total	**-.189**	**-.280, -.097**
Total indirect^a^	-	-
Direct	**-.189**	**-.280, -.097**
Predictor variable: *Emotional Stability*	β	95% CI
Total	**-.468**	**-.547, -.389**
Total indirect^a^	**-.176**	**-.240, -.112**
DT Appraisal	**-.176**	**-.240, -.112**
Direct	**-.292**	**-.388, -.196**
**Outcome variable:**	***GAD symptoms***	
Predictor variable: *Openness*	β	95% CI
Total	.084	-.002, .170
Total indirect^a^	-	-
Direct	.084	-.002, .170
Predictor variable: *Emotional Stability*	β	95% CI
Total	**-.526**	**-.598, -.455**
Total indirect^a^	**-.240**	**-.317, -.163**
DT Absorption	-.036	-.120, .048
DT Appraisal	**-.204**	**-.284, -.124**
Direct	**-.286**	**-.401, -.172**

*Note*. The reduced model only examined the statistically significant associations found in the fully saturated models. The dash indicates coefficients that were not statistically significant in the full saturated model. SES = Socioeconomic Status. DT = Distress Tolerance. GAD = Generalized Anxiety Disorder. Significant associations (*p* < .05) are in bold.

^a^ Reflects the combined indirect associations within the model.

#### Depression model

**Fig 2:**
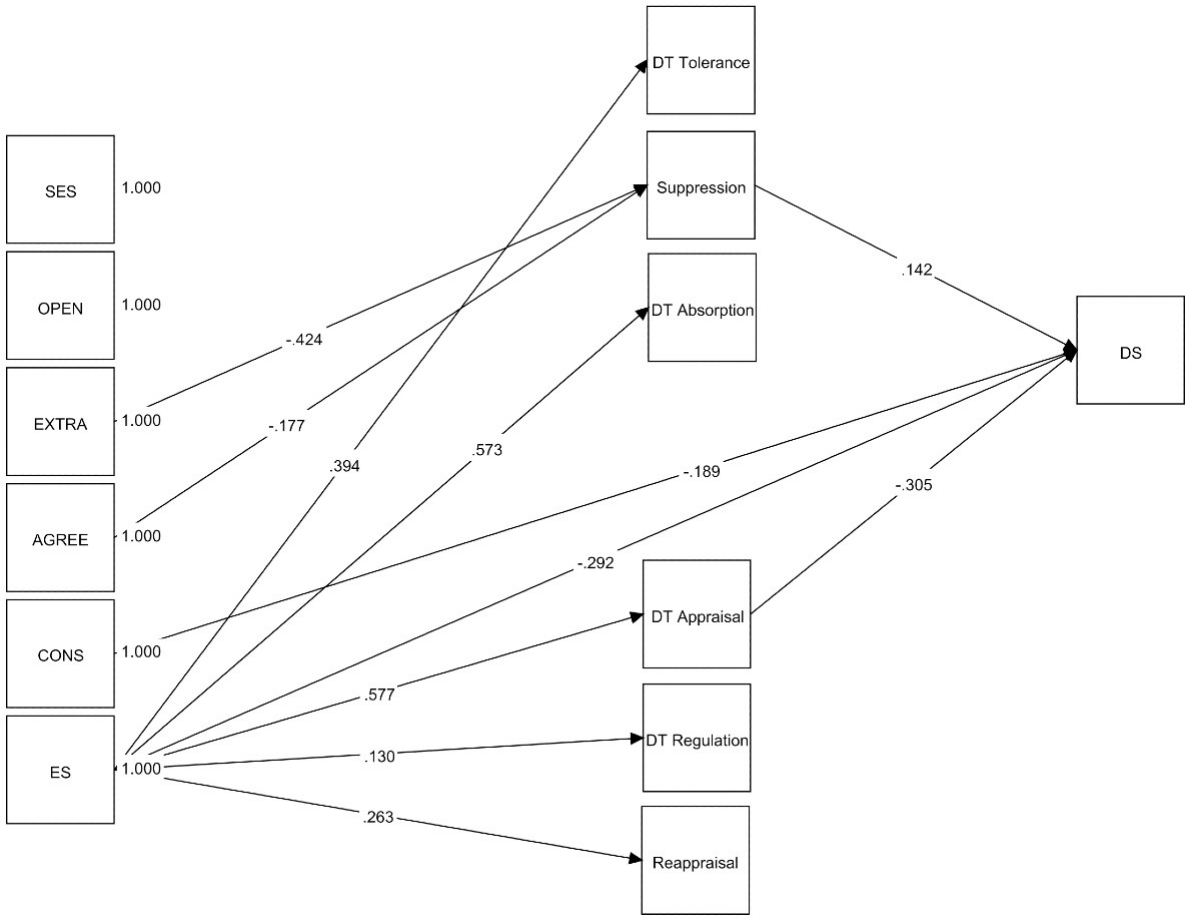
Depiction of the significant standardized effects of the mediation model for depression symptoms. SES: Socioeconomic status; OPEN: Openness; EXTRA: Extraversion; AGREE: Agreeableness; CONS: Conscientiousness; ES: Emotional stability; DT: Distress Tolerance; DS: Depression symptoms. Non-significant path coefficients are not shown in the figure for reasons of parsimony.

The model fit indices indicate an excellent fit to the observed data (χ^2^ (31) = 47.778, *p* = .0276, CFI = 0.985, TLI = 0.970, RMSEA = 0.039, SRMR = 0.036). Fig 2 shows the significant direct associations. Bivariate correlations between the variables included in this model are presented in S1 Table.

SES and openness did not show significant associations with severity of depression. Conscientiousness and emotional stability, on the other hand, were directly associated with depression. Extraversion and agreeableness were indirectly negatively associated with depression via expressive suppression. That is, higher levels of extraversion and agreeableness were associated with a lower tendency to inhibit or reduce the expression of ongoing emotions, which in turn was associated with lower severity of depression. Additionally, emotional stability had an indirect negative association with depression symptoms via appraisal. That is, a higher level of emotional stability was associated with a greater perception of the emotional situation as tolerable which in turn was associated with lower severity of depression.

#### GAD model

**Fig 3:**
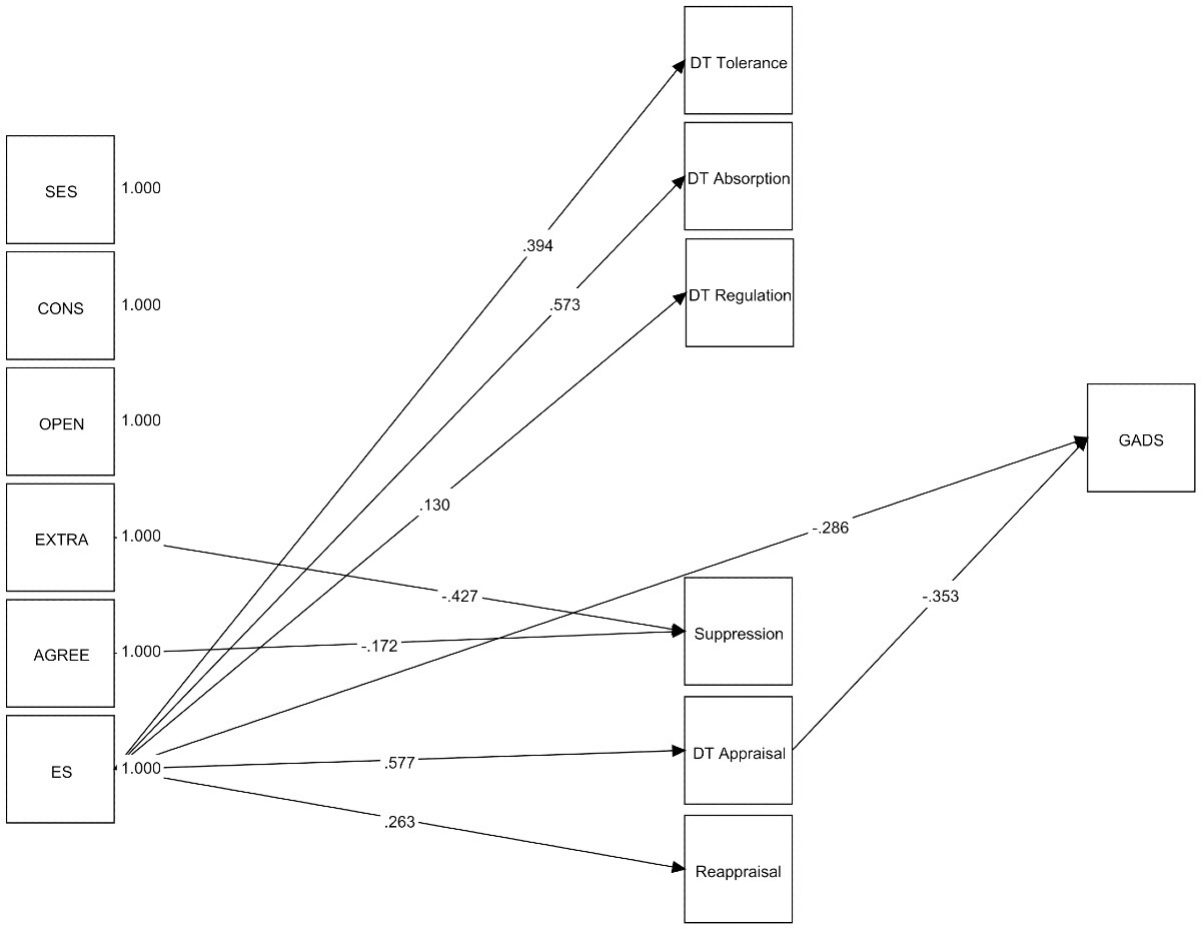
Depiction of the significant standardized effects of the mediation model for GAD symptoms. SES: Socioeconomic status; CONS: Conscientiousness; OPEN: Openness; EXTRA: Extraversion; AGREE: Agreeableness; ES: Emotional stability; DT: Distress Tolerance; GADS: Generalized anxiety disorder symptoms. Non-significant path coefficients are not shown in the figure for reasons of parsimony.

The model fit indices indicate an excellent fit to the observed data (χ^2^ (33) = 52.946, *p* = .0153, CFI = 0.982, TLI = 0.961, RMSEA = 0.042, SRMR = 0.038). Fig 3 shows the significant direct associations. Bivariate correlations between the variables included in this model are presented in S2 Table.

Openness did not show a significant association with severity of GAD. Emotional stability, on the other hand, was directly and indirectly (i.e., via appraisal) associated with GAD symptoms. That is, a higher level of emotional stability was associated with a greater perception of the emotional situation as tolerable which in turn was associated with lower severity of GAD.

## Discussion

The first aim of the study was to describe the occurrence of GAD and depression symptoms in a sample of Argentinian college students. Two findings -which overall fit with previous research- are worth highlighting. First, a relatively high percentage of students endorsed moderate to severe symptoms of depression or anxiety [1, 3, 57]. At a descriptive level, the percentage of students reporting symptoms of depression (45.1%) is larger than the percentage of students reporting symptoms of anxiety (25.9%; [5, 8, 58]). These figures are higher than those from previous research, which also focused on Argentinian college students [8]. That research reported a 38.53% endorsement of symptoms of depression and a 24.65% endorsement of symptoms of anxiety, respectively. Although these disparities could be associated with methodological issues (i.e., different measures to assess symptoms across studies), the key finding is that a sizeable proportion of students is facing these problems. Notably, data for the present study was collected in 2021, during the COVID-19 pandemic. This historical event, associated with increased stress and social isolation levels [59], may have contributed to the relatively high prevalence of depression and anxiety symptoms, reported in this study. Additionally, during this period, classes were still predominantly conducted in a virtual setting rather than on campus, potentially further exacerbating feelings of isolation and affecting mental health. Given previous research indicating that certain personality traits positively impact students’ satisfaction with online teaching methods [60], it is imperative to delve deeper into understanding the potential protective effects these traits may have against heightened psychological distress.

Our second aim was to examine the associations of SES and personality traits with symptoms of depression and GAD via ER and DT. Unlike past research [43, 44], we did not find an association between SES and mental health problems in our reduced model. The limited variability in SES in our sample may explain this result. The student population enrolled in Argentinian public universities largely consists of students coming from middle to high socioeconomic backgrounds [45].

As hypothesized and in line with previous research [8], emotional stability was negatively associated with symptoms of depression and GAD. Specifically, we found that low emotional stability was directly and indirectly associated with more symptoms of depression and anxiety. The indirect association, via appraisal -a DT component-, suggests that individuals with high tendency to feel intense negative emotions and low self-esteem may perceive emotional situations as more distressing, contributing to higher levels of depression and anxiety symptoms. The heightened vulnerability of individuals demonstrating low emotional stability (i.e., high neuroticism) could potentially be mitigated by focusing on DT, a trans-diagnostic construct that significantly influences affective disorders [39]. Interventions targeting improvements of DT have demonstrated promising efficacy in reducing substance use [61] and addressing mental health problems [62]. Such interventions may prove especially beneficial for vulnerable populations such as college students, and particularly for those characterized by high levels of neuroticism.

We found partial support for the hypothesis positing that low extraversion would be indirectly associated with more symptoms of depression and anxiety via ER and DT. Extraversion was significantly and negatively associated with severity of depression, but not anxiety, via expressive suppression (a component of ER). Neither cognitive reappraisal nor DT mediated this association. This suggests that individuals with low levels of sociability and less tendency to seek exciting activities may be more prone to avoid the expression of their emotions, which is associated with more depressive symptoms [8, 9]. We did not find a significant association between extraversion and GAD. It is worth noting that a meta-analysis showed that the negative association between extraversion and anxiety disappeared after controlling for the effect of emotional stability [9]. This suggests that the correlation between extraversion and GAD might be influenced by the shared variance with emotional stability, which could explain why, in our multivariate model, this association was not statistically significant. Contrary to what was proposed in our third hypothesis (that lower conscientiousness would be associated with more symptoms of depression and anxiety indirectly through cognitive reappraisal), lower conscientiousness had a direct positive association with symptoms of depression. This suggests that individuals with lower levels of conscientiousness, characterized by limited self-discipline, responsibility and organization, are more likely to experience depressive symptoms, but this vulnerability does not operate through ER. Other variables related to health risk behaviors such as physical inactivity or unhealthy eating and drinking habits [63, 64] should be explored as potential mediators between this trait and depression symptoms.

An unexpected finding from our exploratory model, that nonetheless resembles previous findings [11, 65], was the positive and negative direct associations of openness with symptoms of anxiety and depression, respectively. However, these associations were not replicated in our reduced model, a finding that is in line with a previous meta-analysis [8] where openness was largely unrelated to several pathologies including depression, anxiety, and substance use disorders. Another noteworthy finding, which was not hypothesized, pertained to the negative indirect association between agreeableness and depression via ER. This suggests that individuals with higher agreeableness, characterized by traits like empathy and cooperation, may be less prone to use expressive suppression. Their tendency toward positive social interactions could reduce the need for suppressing emotions, potentially serving as a protective factor against depression symptoms.

Regarding the mediating role of DT, consistent with Sandín et al. [30], we found that DT mediated the association between emotional stability and symptoms of depression and anxiety. Specifically, it seems that students who exhibit higher levels of emotional stability perceive themselves as more capable of tolerating distress, which in turn is associated with the experience of fewer symptoms of mental health problems. By including the four subcomponents of DT, rather than a global measure [30], the present study provides a more nuanced understanding of the mechanisms underlying this association. Specifically, in our study, emotional stability was associated with all four subcomponents of DT, but only appraisal was associated with fewer symptoms of depression and GAD, suggesting that the ability to evaluate an emotional situation as acceptable plays a key role in protecting college students from developing symptoms of internalizing mental health problems. Noteworthy, although absorption (i.e., being able to not concentrate most of one’s attention on the experience of distress) emerged as a significant mediator in our exploratory model, this pattern of associations was not confirmed in the reduced model. Recent findings from the alcohol field reported that appraisal and absorption were uniquely associated with alcohol consumption via coping drinking motives (i.e., consuming to alleviate negative affect) in a sample of Argentinian college students [49]. Although more studies are necessary to determine the relevance of these DT facets as protective factors for mental health, altogether, these results highlight the role of DT, particularly appraisal and absorption, to explain internalizing and externalizing mental health problems. Colleges should implement procedures aimed at identifying students experiencing mental health issues and help them find proper assistance. The implementation of procedures aimed at increasing DT and an efficient management of negative affect could be a promising venue of intervention.

Unexpectedly, ER did not mediate the association between emotional stability or extraversion and symptoms of depression or anxiety. Emotional stability was the only personality trait associated with higher use of cognitive reappraisal; however, we did not find an association between cognitive reappraisal and mental health symptomatology. This contrasts with previous findings where high extraversion was associated with less anxiety symptoms via cognitive reappraisal [22]. Cognitive reappraisal’s lack of mediating role in this study is surprising but it underscores the complex nature of ER, where the adaptiveness of a strategy may vary depending on the context in which it is implemented. Cognitive reappraisal may be less beneficial in contexts where the intensity of the emotional stimuli is high, or in situations where individuals have chronically low levels of control [41].

Expressive suppression, in turn, significantly mediated the associations between agreeableness/extraversion and symptoms of depression. This is an interesting finding given that these traits are associated with social behaviors, and the use of expressive suppression has been found to predict lower levels of social support, reduced closeness to others, and reduced social satisfaction [66]. For people low on agreeableness and extraversion, employing this ER strategy could entail additional social costs, potentially contributing to the exacerbation of their depressive symptoms. These results, in accordance with previous findings, indicate that expressive suppression is a predominantly maladaptive strategy, mainly associated with negative consequences [17–19, 25].

### Limitations and future directions

The results of the present study should be interpreted considering a number of limitations. First, we used a convenience sampling procedure and the final sample did not have an adequate balance in terms of sex and was predominantly composed by women (81.4%). The invitation to participate in the study was mainly disseminated through social networking sites, which could represent a bias towards those students who use them. Additionally, data was collected between March and April 2021, which overlaps with the COVID-19 pandemic. At that time, however, the Argentinian government had significantly eased the restriction measures to prevent the spread of that disease. All these factors might have reduced the external validity of our findings, which limits the possibility of generalizing them to the population of college students in Argentina. Furthermore, the cross-sectional design prevents us from establishing temporal influences between the variables. Longitudinal studies are needed to evaluate the temporal mediation of distress tolerance and levels of expressive suppression in the association between personality traits and negative affect. Future studies may consider exploring a broader range of emotion regulation strategies beyond those examined in our study, such as acceptance or mindfulness-based approaches, to elucidate their potential mediating effects on the relationship between personality traits and mental health outcomes among college students.

## Conclusions

Beyond these limitations, the present study improves our understanding of the development and maintenance of internalizing mental health problems in emerging adulthood. Taken together, the results of this study suggest that higher levels of emotional stability, extraversion, and agreeableness could protect students from the development of symptoms of depression/anxiety via lower maladaptive ER strategies and higher DT. Particularly, emotional stability emerged as the personality trait consistently related with both depression and anxiety symptoms via difficulties in affect management. DT and ER, compared to personality traits, are variables more amenable to be modified by interventions. Short programs, such as mind-body skills training [36] and compassion cultivation training [67], have yielded increases in DT and decreases in the use of expressive suppression, respectively. These changes, in turn, were associated with mental health improvements. The present study highlights the relevance of developing, for those undergoing internalizing mental health problems, intervention strategies specifically tailored to improve DT and the use of adaptive ER strategies.

## Supporting information

S1 Table Bivariate correlations between depression symptoms and variables included in the reduced model. (DOCX)

S2 Table Bivariate correlations between GAD symptoms and variables included in the reduced model. (DOCX)
