# Supplementary material for: Association between personality traits and symptoms of depression and anxiety via emotional regulation and distress tolerance
Source: PLoS One. 2024 Jul 18;19(7):e0306146. doi: 10.1371/journal.pone.0306146 (PMC11257275; doi:10.1371/journal.pone.0306146)
Supplement: S1 Table — (DOCX) [file pone.0306146.s001.docx]

**S1 Table.**

*Bivariate correlations between depression symptoms and variables included in the reduced model*

|  | 1 | 2 | 3 | 4 | 5 | 6 | 7 | 8 | 9 | 10 | 11 | 12 |
| --- | --- | --- | --- | --- | --- | --- | --- | --- | --- | --- | --- | --- |
| 1. SES |  |  |  |  |  |  |  |  |  |  |  |  |
| 2. Openness | .06 |  |  |  |  |  |  |  |  |  |  |  |
| 3. Extraversion | .08 | **.29** |  |  |  |  |  |  |  |  |  |  |
| 4. Agreeableness | .08 | **.20** | **.18** |  |  |  |  |  |  |  |  |  |
| 5. Conscientiousness | **.17** | .07 | **.19** | **.17** |  |  |  |  |  |  |  |  |
| 6. Emotional Stability | **.16** | **.16** | **.32** | **.21** | **.29** |  |  |  |  |  |  |  |
| 7. DT Tolerance | .04 | .09 | **.15** | **.11** | **.19** | **.44** |  |  |  |  |  |  |
| 8. DT Absorption | **.14** | .08 | **.23** | **.12** | **.24** | **.59** | **.64** |  |  |  |  |  |
| 9. DT Appraisal | **.18** | **.16** | **.26** | **.17** | **.24** | **.56** | **.53** | **.61** |  |  |  |  |
| 10. DT Regulation | .03 | .06 | -.03 | .03 | .06 | **.12** | **.35** | **.15** | **.30** |  |  |  |
| 11.Cognitive Reappraisal | .00 | **.18** | **.21** | **.13** | **.17** | **.30** | .10 | **.26** | **.23** | -.07 |  |  |
| 12. Expressive Suppression | .01 | **-.15** | **-.39** | **-.31** | -.06 | **-.13** | -.07 | **-.12** | **-.20** | -.02 | .08 |  |
| 13. Depression Symptoms | **-.22** | -.02 | **-.27** | **-.20** | **-.33** | **-.54** | **-.37** | **-.47** | **-.48** | -.07 | **-.16** | **.24** |

*Note.* Significant correlations (p ≤ .05) are in **bold** typeface for emphasis. SES = Socioeconomic status. DT = Distress tolerance.
